# Structural Engineering of Tyrosine-Based Neuroprotective Peptides: A New Strategy for Efficient Blood–Brain Barrier Penetration

**DOI:** 10.3390/foods14213744

**Published:** 2025-10-31

**Authors:** Zehui Li, Qiyue Zhu, Yashu Qiao, Junxi Fu, Li Tao, Weihong Min

**Affiliations:** 1 National Key Laboratory for Development and Utilization of Forest Food Resources, Zhejiang A&F University, Hangzhou 311300, China; lizehui063@stu.zafu.edu.cn (Z.L.); zhuqiyue1116@stu.zafu.edu.cn (Q.Z.); qiaoyashu@stu.zafu.edu.cn (Y.Q.); fujunxi0820@stu.zafu.edu.cn (J.F.); taoli@zafu.edu.cn (L.T.); 2 College of Food Science and Engineering, Jilin Agricultural University, Changchun 130118, China; 3 College of Food and Health, Zhejiang A&F University, Hangzhou 311300, China; 4 Provincial Key Laboratory for Non-Wood Forest and Quality Control and Utilization of Its Products, Zhejiang A&F University, Hangzhou 311300, China

**Keywords:** blood–brain barrier, walnut-derived peptide, secondary structure, tyrosine, antioxidant

## Abstract

The relationship between the structure of walnut-derived peptides and their activity of transport efficiency across the blood–brain barrier (BBB) remains unclear. In this study, a series of walnut-derived peptides were synthesized by substituting leucine (L) with tyrosine (Y), lysine (K), or arginine (R). Three outstanding peptides—EVSGPGYSPN, TWLPYPR, and YVPFPYP—were selected based on their antioxidant capacity and BBB transport efficiency, with EVSGPGYSPN exhibiting the highest activity. Reversed-phase high-performance liquid chromatography (RP-HPLC) and Transwell assay results demonstrated that EVSGPGYSPN can remain stable during gastrointestinal digestion and penetrate the BBB. Pharmacokinetic results revealed that the cumulative concentration of EVSGPGYSPN in the brain reached 1.25 ± 0.91 µg/g at 10 h, while its plasma half-life exceeded 12 h. Furthermore, it significantly reduced reactive oxygen species (ROS) levels to 110.46 ± 15.16%. Nuclear magnetic resonance (NMR) and Fourier-transform infrared spectroscopy (FTIR) results indicated that EVSGPGYSPN is rich in aromatic hydrogen signals and exhibits low methyl signals, which may enhance its antioxidant activity. Circular dichroism (CD) spectroscopy showed that EVSGPGYSPN has the highest random coil content, which facilitates its binding to transporters on the BBB and promotes BBB permeability. This study provides valuable insights into the design of brain-targeted peptide delivery systems.

## 1. Introduction

Food-derived bioactive peptides, when used as dietary supplements, regulate brain function and significantly improve cognitive performance [1]. However, most of these peptides remain active primarily in the peripheral circulatory system and have limited ability to reach the brain, as peptides do not easily penetrate the blood–brain barrier (BBB) [2]. The BBB is a protective physical barrier formed by tight junctions between brain capillary endothelial cells, preventing foreign bodies in peripheral blood from entering the brain while maintaining cerebral homeostasis [3]. Simultaneously, it restricts the penetration of most functional factors into the central nervous system, preventing them from reaching effective concentrations. To address this challenge, many researchers have focused on local delivery of functional factors using certain invasive methods, including focused ultrasound [4], arterial injection [5], and electron tomography [6], which induce transient and certain openings of the BBB, thereby enabling increased absorption of functional compounds compared to normal brain tissue and facilitating localized delivery. However, these methods also allow blood-borne substances to enter the brain, which can lead to irreversible side effects. Non-pharmacological interventions, including diet and exercise, are the most essential non-invasive strategies for preventing and alleviating brain diseases, owing to their favorable safety profile and minimal side effects [7]. In recent years, food-borne active peptides have attracted more and more attention because of their good functional activity, safety and non-toxicity, and wide sources [8]. The structure of these peptides directly determines their ability to penetrate the BBB.

Researchers have concluded that certain amino acid residues and sequences may affect the secondary structure and physicochemical properties of the peptide, which then affect their ability to penetrate the BBB. Peptides rich in arginine (R) residues, such as TAT(YGRKKRRQRRR) [9], SynB3 (RRLSYSRRRF) [10], and Protamine (VSRRRRRRGGRRRRK) [11], are typical BBB-penetrating peptides, as helicity and hydrophobicity enhance their membrane penetration [12]. Additionally, short proline (P)-rich peptides, known for their neuroprotective functions, have been reported to penetrate the BBB. Related studies also show that the amphiphilic peptide VRLPPP exhibits high BBB penetration ability and biosafety [13,14]. P-rich cationic antimicrobial peptides, including oncocin and apidaecin, penetrate the BBB without disrupting tight junctions between brain endothelial cells [15]. This ability is attributed to P being the only amino acid with a secondary amine and a cyclic side chain, consequently limiting hydrogen bonding [16]. The redox reactions of Y residues are involved in numerous biological processes by mediating proton-coupled electron transfer [17]. However, reports on Y-and Lysine (K)-rich BBB-penetrating peptides remain relatively limited. Only the endogenous neuropeptide analog YK^1^ and neurotensin-derived peptide KKPYILKKKPYIL exhibit analgesic effects via passive transport penetration of the BBB [18]. The peptide RKKYKYRRL not only penetrates the BBB but also protects its barrier function [19]. Therefore, exploring its BBB penetration ability and secondary structure changes through these site-specific amino acid substitutions provides a promising approach for designing brain-targeted delivery peptides.

Recent studies on walnut-derived bioactive peptides reveal their diverse physiological functions, including enhancing learning and memory abilities [20], maintaining gut microbiota [21], and providing antioxidant effects [22]. Previous research on the physiological activity of the walnut peptide EVSGPGLSPN, isolated and purified from a <3 kDa walnut peptide, demonstrates its ability to reduce oxidative stress by inhibiting the NF-κB/caspase inflammatory pathway [23]. When the amino acid at position 7 is replaced with Y, the walnut-derived peptide EVSGPGYSPN successfully crosses the in vitro BBB model via a Caveolin (Cav)-dependent endocytotic pathway [24]. Additionally, the walnut peptide TWLPLPR protects BBB integrity by inhibiting MMP-9 [25] and remains intact in the bloodstream following gastrointestinal digestion, effectively penetrating the BBB from plasma [26]. This alleviates cognitive deficits induced by insulin resistance [27]. Walnut peptide YVPFPLP forms a stable complex with PPARγ to improve glutamate-induced excitotoxicity [28]. Overall, the neuroprotective effects of peptides EVSGPGLSPN, TWLPLPR, and YVPFPLP have been validated in different in vivo models. Based on these findings, this study used these three peptides as the basis for structural modification to explore the relationship between their structure and trans-BBB transport ability.

In this study, structural modifications were performed on walnut peptide EVSGPGLSPN by systematically replacing each amino acid site with P, R, Y and K. Molecular docking was used to screen active peptides with high affinity for Cav, and it was found that EVSGPGYSPN had the highest binding force to Cav. Subsequently, we replaced the seventh amino acid with the other 19 common amino acids and screened the active peptides EVSGPGKSPN and EVSGPSRSPN with high affinity for Cav using molecular docking. Circular dichroism (CD), nuclear magnetic resonance (NMR), and Fourier-transform infrared spectroscopy (FTIR) were employed to analyze the differences in the secondary structures of these peptides. Their abilities to scavenge ABTS, DPPH, and oxygen-free radicals were also evaluated. The transport capacity of these EV peptides across the BBB and their accumulation levels in the brain were assessed using in vitro and in vivo BBB models. The influence of in vivo absorption, metabolism, and systemic circulation on brain targeting was investigated through simulated gastrointestinal digestion experiments and plasma half-life measurements. Finally, using the walnut-derived peptides TWLPLPR and YVPFPLP as models, the tyrosine-containing peptides were validated for their BBB-transport capability and neuroprotective properties. This study provides novel structural insights for designing brain-targeting peptide delivery systems.

## 2. Materials and Methods

### 2.1. Chemicals and Reagents

The walnut-derived peptides were synthesized by Jiangsu Ji Tai Peptide Industry Science and Technology Ltd. (Yancheng, China) with a purity of more than 97% and desalinated. The mouse line HT22 was obtained from Zhong Qiao Xin Zhou Biological Technology Ltd. (Shanghai, China). Dulbecco’s modified Eagle’s medium (DMEM) was purchased from HyClone Co. (Logan, UT, USA). Fetal bovine serum (FBS), 0.25% pancreatin with ethylenediaminetetraacetic acid (0.25% EDTA), and a penicillin/streptomycin mixed solution were acquired from Meilun Biotechnology Co., LTD (Dalian, China).

### 2.2. Molecular Docking

The Cav molecular structure was predicted utilizing Alphafold2, and the EV peptide structure was generated using Discovery Studio 2019. ADCP was employed to calculate the docking of Cav with 20 EV peptide molecules, and LigPlus was utilized to analyze the interaction between Cav and EV peptides. Additionally, Pymol was deployed for visualization. The binding affinity of the peptide to Cav was assessed based on binding energy, with the results expressed in kcal/mol.

### 2.3. NMR Measurements

The ^1^H NMR spectra were obtained utilizing a Bruker Avance 600 MHz nuclear magnetic resonance spectrometer (Bruker BioSpin GmbH, Rheinstetten, Germany) equipped with a 5 mm broadband fluorine observation probe (BBFO) with z-gradient. The peptides were each dissolved in 600 µL of D_2_O for analysis, and ^1^H NMR spectra were recorded. The main experimental parameters included a sampling delay time of 6 s and a pulse width of 13 µs, with a total of 32 scans.

### 2.4. FTIR Spectroscopy

Peptides were analyzed employing FTIR spectroscopy (Thermo Fisher Scientific, Waltham, MA, USA). Each peptide was mixed with 200 mg KBr powder and then pressed into pellets for measurement. The measurement ranged from 400−4000 cm^−1^.

### 2.5. CD Measurements

The circular dichroism spectra of the peptides were measured utilizing a JASCO J-820 circular dichroism spectrometer (JASCO Corp., Tokyo, Japan). A peptide solution at a concentration of 0.5 mg/mL was placed in a quartz disc with a 1 nm bandwidth. The spectral detection ranged from 190 to 260 nm, with a scanning speed of 100 nm/min. Each sample was scanned thrice with a response time of 2 s. The method for calculating the secondary structure follows the procedure described by Ding et al. [29].

### 2.6. Antioxidant Activity of EV, TW, and YV Peptides

#### 2.6.1. Determination of DPPH Radical Scavenging Activity

A modified DPPH scavenging assay was conducted following the method described by Shen et al. [30]. 0.0023 g of DPPH was mixed with 100 mL of 95% absolute ethanol to prepare a 60 µmol/L DPPH radical working solution. The sample was then mixed with 60 µmol/L DPPH free radical working solution at a 1:1 (*v*/*v*) ratio, mixed thoroughly, and allowed to stand in the dark for 30 min. The mixture was then centrifuged at 4500 rpm for 10 min. The absorbance was measured at a wavelength of 517 nm and compared with an equivalent concentration of GSH. The DPPH radical scavenging rate was calculated using the following formula:

(1) $$\mathrm{DPPH}\;\mathrm{radical}\;\mathrm{scavenging}\;\mathrm{ratio}\;\%\;=\;1-\frac{A_{a}-A_{b}}{A_{c}}\times 100\%$$

where A_a_ represents the absorbance value of EV, TW and YV peptides solution added to DPPH scavenging radical working solution; A_b_ denotes the absorbance value of the solution with 95% (*v*/*v*) absolute ethanol and the ABTS scavenging radical working solution; A_c_ is the absorbance value after mixing distilled water and DPPH in equal volumes of 1:1.

#### 2.6.2. Determination of ABTS Radical Scavenging Activity

ABTS scavenging radical determination was conducted following the procedure of Li et al. with slight modifications [31]. A 7 mmol/L aqueous solution of ABTS and a 2.49 mmol/L aqueous solution of potassium persulfate were mixed in a 1:1 ratio. The mixture was allowed to stand in the dark at 4 °C for 12–16 h. The concentration was adjusted using 5 mmol/L PBS (pH 7.4) to ensure that the absorbance of the ABTS radical stock solution was 0.700 ± 0.002 at a wavelength of 734 nm. EV and TW peptide aqueous solutions (10 µL), each at a concentration of 1 mg/mL, were taken. A glutathione aqueous solution with the same substrate concentration was used as the control group. After adding the samples to a black 96-well plate, 190 µL of ABTS free radical working solution was added to each well. The absorbance at 734 nm was measured using a microplate reader after a 6 min reaction.

The ABTS clearance was calculated as follows:

(2) $$\mathrm{ABTS}\;\mathrm{radical}\;\mathrm{scavenging}\;\mathrm{rate}\;\%\;=\;1-\frac{A_{a}-A_{b}}{A_{c}}\times 100\%$$

where A_a_ represents the sample; A_b_ represents the absorbance of the solution in which the ABTS working solution was substituted with 5 mmol/L PBS; A_c_ represents the absorbance of the blank group.

#### 2.6.3. Oxygen Radical Absorbance Capacity (ORAC) Assay

ORAC assay was performed based on the technique of Simon et al., with minor modifications [32]. First, in black 96-well plates, 25 µL of PBS buffer (75 mmol/L, pH 7.4), 25 µL of Trolox standard solutions (at concentrations of 6.25 µM, 12.5 µM, 25 µM, 50 µM), and the peptide solution were added at a 1:1 volume ratio. Then, the samples were incubated at 37 °C for 10 min. Subsequently, 150 µL of 63 mmol/L sodium fluorescein was added to all wells and incubated at 37 °C for 20 min. Following this, 25 µL of 6 mmol/L azobisisobutyramidine hydrochloride (AAPH) solution was quickly added to each well. Two control groups were established: one consisted of a black 96-well plate with free radicals (+AAPH), while the other group included a black 96-well plate without free radicals (−AAPH). Both plates were placed in a microplate reader and shaken for 5 s. The excitation wavelength was set to 485 ± 20 nm, and the emission wavelength was set to 530 ± 10 nm. The entire system was set at 37 °C, and the fluorescence intensity was measured every 2 min. The area under the fluorescence decay curve (AUC) was calculated utilizing the approximate integration method as follows:

AUC = [2 × (*f*_0_ + *f*_1_ ⋯ + *f*_k−1_ + *f*_k_) − *f*_0_ − *f*_k_] × △t × 0.5 (3)

where f_k_ represents the relative fluorescence intensity at the nth measurement point, and Δt is the interval time of 2 min between adjacent measurement points.

The measurement results were expressed as ORAC values using the following formula:

ORAC value = [(AUC_peptide_ − AUC_blank_)/(AU_trolox_ − AUC_blank_)] × C_trolox_/C_peptide_ (4)

The ORAC value of the sample was expressed in µmol Trolox equivalent/g (µmolTE/g).

### 2.7. Cell Culture and Treatment

Mouse brain microvascular endothelial cells (bEnd.3) and mouse hippocampal neuron cells (HT22) were purchased from Zhong Qiao Xin Zhou Biological Technology Ltd. (Shanghai, China). Cells were cultured in DMEM (Cat # 11965092, Gibco, New York, USA) supplemented with 1% (*v*/*v*) penicillin/streptomycin (MA0110, MeilunBio, Dalian, China) and 10% (*v*/*v*) fetal bovine serum (C04001-500, Vivacell, Shanghai, China) in a 5% CO_2_ humidified incubator at 37 °C, and passaged every 2 days. When cell confluence reached 85–90%, cells were seeded into 6/12 well plates at a density of 1 × 10^5^ cells/mL. Once the confluence reached 85–90% again, the cells were cultured in H_2_O_2_ medium at varying concentrations for 2 h as a model group. Cell viability was assessed using MTT at 0.1 mg/mL, and the optimal H_2_O_2_ concentration was determined when the cell viability approached 50%.

When cell confluence reached 80–90%, they were incubated with 100 µM EV peptides and TW peptides, respectively, for 24 h, followed by injury with 800 µM H_2_O_2_ for 2 h to observe the protective effect of the peptides. Among them, the model group was directly injured after 24 h of incubation with DMEM.

### 2.8. Transwell

The method described by Han et al. was referenced and modified [33]. Briefly, 500 µL of bEnd.3 cells at a density of 5 × 10^4^ cells/mL were seeded in the upper chamber of Transwell (0.4 µm, 12-well, PET material), and 1.5 mL of DMEM was added to the lower chamber to ensure the upper and lower chambers were aligned. After cell adherence, the culture medium in the upper chamber was changed daily, while the culture medium in the lower chamber was changed every other day. Once the transendothelial electrical resistance (TEER) was > 200 Ω·cm^2^, both the upper and lower chambers were replaced with serum-free DMEM. After 12 h, EV and TW peptides, solubilized in HBSS at a concentration of 100 µM, were added to the upper chamber. After 4 h, the samples were collected separately, and RP-HPLC detection, along with Origin 2021 mapping analysis, was performed. TEER and apparent permeability coefficient (Papp) were calculated as follows:

TEER = (TEER_sample_ − TEER_control_) × Cell Culture Area (5)

(6) $$\mathrm{Papp}=\frac{∆C\cdot V}{∆t\cdot C_{0}\cdot A}(cm/s)$$

where ΔC represents the concentration in the lower chamber (µM), V is the volume of the lower chamber (mL), Δt indicates the experimental time (s), C_0_ is the initial concentration in the upper chamber, and A is the membrane area of the Transwell (cm^2^).

### 2.9. Real-Time Ex Vivo Imaging of Rhodamine B-EV Peptide Injected into the Tail Vein

This study was approved by the Animal Experiment Ethics Committee of Zhejiang A&F University under ethics number ZAFUAC202413. C57/BJ mice were purchased from SPF Biotechnology Co., Ltd. (Suzhou, China). and housed at a constant temperature of 23 ± 2 °C for 12 h. After 1 week of acclimation, the mice were injected via the tail vein with rhodamine B-EVSGPGLSPN and Rhodamine B-EVSGPGYSPN (60 mg/kg body weight). The mice were sacrificed at 1, 4, 8, and 12 h, respectively, and their brains were collected for ex vivo fluorescence imaging employing an IVIS Lumina XR system.

### 2.10. In Vitro Gastrointestinal Digestion

Walnut peptides were dissolved in distilled water to prepare a 100 µM aqueous solution. The pH was adjusted to 2.0, and pepsin was added to simulate gastric juice digestion. After 1 and 2 h in a 37 °C water bath, 1 mL of the sample was taken and stored at 4 °C for subsequent use. The pH of the remaining solution was then adjusted to 7.0, and 5 mg/mL trypsin was added to simulate intestinal digestion. After 1 and 2 h in a 37 °C water bath, 1 mL samples were taken, filtered through a 0.22 µm pore size membrane, and analyzed via RP-HPLC.

### 2.11. Plasma Half-Life

The plasma half-life was evaluated utilizing KM mice (20 ± 2 g) obtained from Changsheng Biotechnology Co., Ltd. (Benxi, Liaoning, China). After the adaptation period, the mice were euthanized by cervical dislocation. Blood was collected from the heart and immediately transferred into an EP tube containing heparin sodium. After standing for 2 h, the plasma was separated by centrifugation at 4500 rpm at 4 °C for 15 min. A 500 µL aliquot of 500 µM peptide solution was added to 1 mL of blank plasma, mixed thoroughly by vortexing, and methanol was added in a 1:3 ratio. After vortexing for 1 min, the mixture was centrifuged at 12,000 rpm for 10 min, and the resulting supernatant was collected for RP-HPLC analysis.

### 2.12. Brain Pharmacokinetics

Six C57/BJ mice were randomly selected, and the peptides EVSGPGLSPN and EVSGPGYSPN were administered via gavage, respectively. After 10 h, the mice were euthanized by cervical dislocation, and the brains were harvested. For analysis, 3 mL of normal saline was added to each gram of brain tissue, which was then homogenized. The sample was diluted in a ratio of 1:3 with methanol solution, mixed thoroughly, and centrifuged at 12,000 rpm for 10 min. The resulting supernatant was collected for RP-HPLC analysis.

### 2.13. RP-HPLC Analysis

After gel filtration chromatography, the most active fractions from the Transwell, plasma, and brain homogenate samples were further separated utilizing a symmetrical C18 column (5 µm, 20 µL load). Gradient elution was conducted with a mobile phase consisting of pure water with 0.1% TFA (mobile phase A) and acetonitrile containing 0.1% TFA (mobile phase B). The elution process for mobile phase B was as follows: EVSGPGLSPN: 10−35%, EVSGPGYSPN: 8−33%, EVSGPGRSPN: 5−30%, EVSGPGKSPN: 4−29%, TWLPLPR: 24−49%, TWLPYPR: 21−46%, TWLPRPR: 16−41%, TWLPKPR: 15−40%, YVPFPLP, YVPFPYP: 11–34%. This was performed for 25 min at a flow rate of 1 mL/min.

Among them, EVSGPGLSPN, EVSGPGRSPN, EVSGPGKSPN, TWLPLPR, TWLPYPS, TWLPRPR, and TWLPKPR were detected using an absorbance detector (Shimadzu LC-20AT HPLC) at 220 nm. EVSGPGYSPN, YVPFPLP, and YVPFPYP were detected using Waters e2695 at 220 nm.

### 2.14. Measurement of Cellular Reactive Oxygen Species (ROS) Levels

HT22 cells were treated as described above and then washed three times with PBS. Cells were incubated with 2′, 7′-dichlorofluorescein diacetate at 37 °C for 20 min in the dark, and images were acquired using an inverted fluorescence microscope (Leica dmi8). The fluorescence intensity was measured at an excitation wavelength of 485 nm and an emission wavelength of 525 nm.

### 2.15. Statistical Analysis

Data analysis was performed using SPSS 20.0 (IBM, New York, NY, USA). Results from at least three independent experiments are expressed as mean ± SD. Statistical significance was assessed by one-way ANOVA followed by Duncan’s test, with a *p* < 0.05 considered significant. All graphs were generated using GraphPad Prism 6 and Origin 2021, respectively.

## 3. Results and Discussions

### 3.1. Construction and Screening of BBB Penetrating Peptides

**Figure 1:**
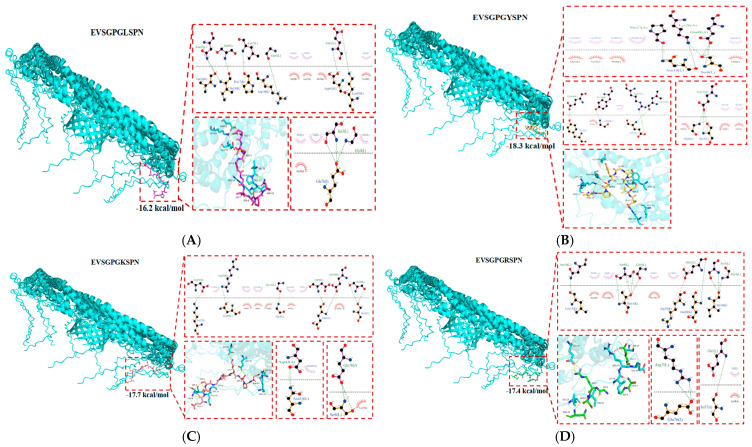
Screening and identification of BBB penetrating peptides. (**A**) EVSGPGLSPN; (**B**) EVSGPGYSPN; (**C**) EVSGPGKSPN; (**D**) EVSGPGRSPN and Cav molecule docking.

Table 1 shows the molecular docking results for the other 19 amino acids substituted at the seventh site of the walnut peptide EVSGPGLSPN with the active region of Cav. The peptides EVSGPGYSPN, EVSGPGKSPN, and EVSGPGRSPN exhibited strong binding to the Cav protein pocket. These peptide segments were deeply inserted, exhibiting the highest binding affinity, with binding energies of −18.3 kcal/mol, −17.7 kcal/mol, and −17.4 kcal/mol, respectively. In contrast, the binding energy of EVSGPGLSPN to Cav was −16.2 kcal/mol. A lower binding energy indicates a more stable protein–substrate complex. Ligand protein complexes are primarily stabilized by hydrophobic and hydrogen-bonding interactions. Regarding binding mode, the amino acid residues of EVSGPGYSPN interacting with the active pocket of the Cav protein included Asn-10, Ser-8, Tyr-7, and Gly-6. Twelve hydrogen bond interactions were observed with the amino acids Ser-8, Asn-10, Tyr-7, and Gly-4, with hydrogen bond distances of 3.03 Å, 2.75 Å, and 2.94 Å, respectively (Figure 1B). For EVSGPGLSPN, the interacting Cav amino acid residues included Lys-65, Asp-61, Glu-76, Gly-6, Val-16, Pro-17, Ile-18, and Arg-19 (Figure 1A). Among them, 10 hydrogen bonds interacted with the side chain residues of Glu-76 and Lys-65, with hydrogen bond distances of 3.13 Å and 2.85 Å, respectively. Figure 1C, D show the binding diagrams of EVSGPGKSPN, EVSGPGRSPN, and Cav. The hydrogen bond distances between EVSGPGLSPN, EVSGPGYSPN, EVSGPGKSPN and EVSGPGRSPN and Cav are relatively short. The average hydrogen bond lengths are 2.88 Å and 2.84 Å, respectively, both of which are shorter than 3.5 Å, typically observed for traditional hydrogen bonds, highlighting their importance in stabilizing peptide molecules within the active pocket of proteins.

In conclusion, the walnut-derived peptides EVSGPGYSPN, EVSGPGKSPN, and EVSGPGRSPN bind to the active site of the Cav target protein, forming stable complexes with this protein. Based on their low binding energies, we selected the walnut peptide EVSGPGLSPN along with EVSGPGYSPN, EVSGPGKSPN, and EVSGPGRSPN for chemical synthesis and further investigation.

### 3.2. Secondary Structure

To elucidate the structural basis of changes in antioxidant activity, CD, FTIR, and NMR were employed to analyze alterations in the secondary structure of these peptides.

#### 3.2.1. Structural Spectroscopic Analysis of the Peptides

**Figure 2:**
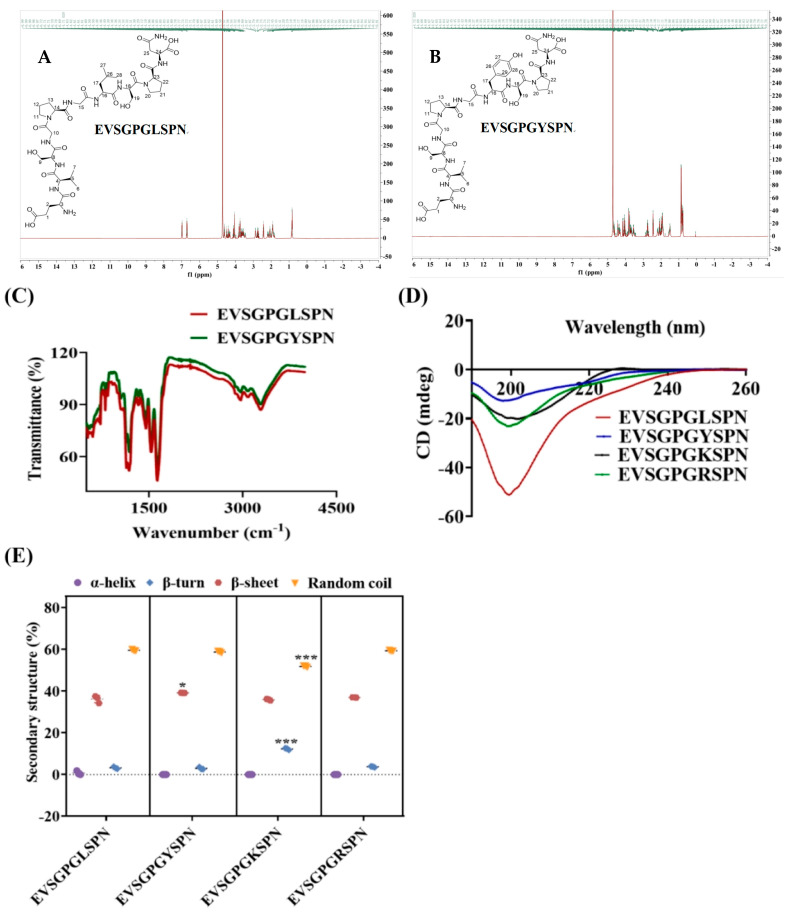
Secondary Structure Analysis of BBB Penetrating Peptides. (**A**,**B**) NMR, (**C**) FTIR, (**D**,**E**) CD of EVSGPGLSPN and EVSGPGYSPN. All experiments were conducted in triplicate (*n *= 3). * *p* < 0.05, *** *p* < 0.001.

Analysis of EVSGPGYSPN and EVSGPGLSPN using nuclear magnetic resonance spectroscopy is a common technique for studying the binding affinity and kinetics of peptide-protein interactions. This technique reveals both the intramolecular and intermolecular interactions of peptide protons. We conducted ^1^H NMR spectroscopic analysis to examine the active hydrogen atoms of the peptides EVSGPGLSPN and EVSGPGYSPN within their respective chemical environments. As shown in Figure 2A,B, a p-hydroxyphenol structure is attached to position 17 of EVSGPGYSPN, whereas the isopropyl structure is attached to peptide EVSGPGLSPN at position 17. Comparative hydrogen spectrum analysis further revealed distinct structural differences between EVSGPGYSPN and EVSGPGLSPN. Analysis of the hydrogen spectrum for EVSGPGYSPN revealed four additional benzene ring hydrogen signals: δH 7.06 (d, J = 8.6 Hz, 2H, H-26,29) and 6.78 (d, J = 8.6 Hz, 2H, H-27,28). For EVSGPGLSPN, an additional isopropyl hydrogen signal was observed: δH 0.94 (d, J = 6.8 Hz, 6H, H-27,28) and 1.67–1.53 (m, 1H, H-17). Next, we analyzed the differences between specific chemical bonds in the two peptides. Figure 2C presents the Fourier infrared spectra of peptides EVSGPGLSPN and EVSGPGYSPN. Both peptides exhibited similar peak shapes, with broad absorption bands for O-H and N-H stretching vibrations observed between 3100 and 3400 cm^−1^, indicating the presence of intermolecular and intramolecular hydrogen bonds. The peak shifts in EVSGPGLSPN and EVSGPGYSPN observed at 2873 cm^−1^ and 2882 cm^−1^ for EVSGPGLSPN and EVSGPGYSPN corresponded to the symmetric or asymmetric stretching of methylene (-CH_2_) and methyl (-CH_3_) groups, respectively. The amide I peak at 1631 cm^−1^ represents the vibrational absorption peak of the amino and carbonyl groups, while the amide II peak at 1533 cm^−1^ represents the vibrational absorption peak of the C-N bond. Notably, an additional 1511 cm^−1^ peak was observed for EVSGPGYSPN relative to EVSGPGLSPN at 1500–1600 cm^−1^, which verified the conclusion of the H-NMR spectroscopy, which may also be caused by the presence of the benzene ring hydrogen signal [34]. This lipid environment prefers hydrophobic molecules to penetrate the BBB rather than hydrophilic molecules [35].

#### 3.2.2. CD

The circular dichroism results are shown in Figure 2D. Circular dichroism analysis revealed a large negative band detected at 200 nm for EV peptides. The α-helix, β-turn, β-sheet, and irregular coiled proportions of the EV peptides were further examined (Figure 2E). The content of β-sheet in EVSGPGYSPN was significantly higher than that of other EV peptides (*p* < 0.05), and the content of irregular coil was the highest. This means that they have a high structural flexibility [36], more adapted to transporters or receptors on the BBB. The amino acid sequence, position of specific amino acids, and peptide chain length are considered key factors influencing the penetration of peptides into the BBB. Previous studies indicate that BBB-penetrating peptides are typically rich in K, R, and H, which can confer higher positive charges to the peptides. These charges promote electrostatic interaction with the negatively charged phospholipid bilayer on the cell membrane, facilitating transmembrane transport [37].

### 3.3. Antioxidant Activity

**Figure 3:**
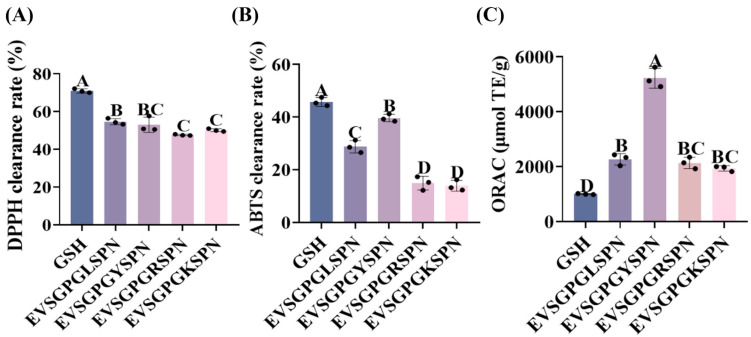
Comparison of Antioxidant Activity among EV Peptides. Scavenging ability of EV peptide to (**A**) DPPH, (**B**) ABTS, (**C**) ORAC. Statistical significance was considered at *p* < 0.05. Dissimilar letters represent significant differences between groups, whereas identical letters indicate no significant difference.

Natural antioxidants help balance free radical production and actively scavenge them in excess, thereby reducing brain damage induced by free radicals [38]. Stable radicals such as DPPH, ABTS, and oxygen radicals are commonly used to assess the ability of natural antioxidants to donate hydrogen atoms and evaluate their antioxidant activity [39]. Therefore, we evaluated EVSGPGLSPN, EVSGPGYSPN, EVSGPGRSPN, and EVSGPGKSPN for their DPPH radical, ABTS radical scavenging ability, and oxygen radical absorption ability, respectively (Figure 3).

#### 3.3.1. DPPH Radical Scavenging

Figure 3A shows the DPPH radical scavenging rates of EV peptides. All screened peptides demonstrated the scavenging ability of DPPH free radicals. At a substrate concentration of 1 mg/mL, the scavenging activities of EVSGPGLSPN, EVSGPGYSPN, EVSGPGRSPN, and EVSGPGKSPN were 54.66 ± 1.61%, 54.13 ± 4.06%, 47.60 ± 0.35%, and 50.13 ± 0.75%, respectively. Among these, EVSGPGLSPN exhibited significantly higher activity than EVSGPGRSPN (*p* < 0.05), while the other three peptides showed no significant difference (*p* > 0.05). The higher DPPH scavenging activity may be attributed to the low molecular weight peptides, as indicated in Table 2, where EVSGPGLSPN has the lowest molecular weight among the EV peptides. This finding aligns with the report by Arise et al., which demonstrates an inverse correlation between the molecular weight of peptides and their DPPH free radical scavenging activity. This is likely attributed to more efficient interactions with free radicals [40]. Furthermore, the radical scavenging ability of DPPH is positively correlated with the proportion of hydrophobic amino acids in the peptide [41]. L has a large hydrophobic side chain, and this side chain structure is more conducive to hydrophobic interaction with DPPH radicals. When the DPPH radical is close to the peptide, the hydrophobic region of the L side chain may encapsulate part of the structure of the DPPH radical, leaving the radical in a relatively restricted microenvironment. Among the screened EV peptides, only EVSGPGLSPN has a hydrophobic amino acid content of 60%, while EVSGPGYSPN, EVSGPGKSPN, and EVSGPGRSPN each have 50%. Therefore, this may be the reason why the DPPH clearance of the L-based peptides is higher than that of other EV peptides.

However, although EVSGPGYSPN exhibits the highest molecular weight, their ability to scavenge DPPH free radicals is not the lowest. This may be as reported by Weng et al., peptides containing a Y residue alongside free Y amino acids are crucial for effective DPPH radical scavenging [42]. Additionally, P and Y residues have been identified as key amino acids for scavenging DPPH radicals [43], as they can provide strong hydrophobic interactions owing to their aromatic nature. And when that peptide sequence contains a large amount of Y, the antioxidant capacity of the peptide is enhanced owing to DPPH lipophilicity [44].

#### 3.3.2. ABTS Radical Scavenging

Figure 3B shows the ABTS radical scavenging rate. EVSGPGYSPN exhibited the highest ABTS radical scavenging activity at a substrate concentration of 1 mg/mL, with values of 39.64 ± 1.43%. Both EV peptides showed lower ABTS radical activity compared to that of DPPH, which may be attributed to the hydrophobic nature of peptides. Among the EV peptides selected, only EVSGPGYSPN is a hydrophobic peptide. However, unlike DPPH radical scavenging activity, EVSGPGYSPN exhibited the highest ABTS radical scavenging activity. This aligns with the findings of Liu et al., revealing that Y-containing peptides demonstrate significant ABTS radical scavenging activity [45]. Aromatic amino acids are considered the most effective free radical scavengers [46]. This is attributed to the unique ability of phenol to donate protons and maintain stability through resonance structure, which allows Y as a peptide component to maximize the peptide antioxidant activity [47].

#### 3.3.3. ORAC

ORAC is commonly utilized to assess the ability of various antioxidants to facilitate hydrogen atom transfer. This technique is particularly relevant to human biology as it can simulate the actions of hydrophilic and hydrophobic antioxidants by disrupting lipid peroxy radical chains involved in the peroxidation reaction of biological components [48]. Figure 3C shows the oxygen radical absorbance capacity of the EV peptides. At a substrate concentration of 1 mg/mL, the ORAC values of EVSGPGYSPN (5221.02 ± 363.35 µmol TE/g) were significantly higher than EVSGPGLSPN (2266.90 ± 207.37 µmol TE/g) (*p* < 0.05). Concurrently, it was significantly higher than the ORAC value of 1003.3 ± 18.6 µmol TE/g for GSH (*p* < 0.05). This is consistent with the ABTS radical scavenging rate, as the synergistic effect between the ABTS free radical scavenging rate and ORAC is the strongest. However, it exhibited a low correlation with DPPH free radical scavenging ability [47].

### 3.4. In Vitro BBB Penetration Ability of EV Peptides

**Figure 4:**
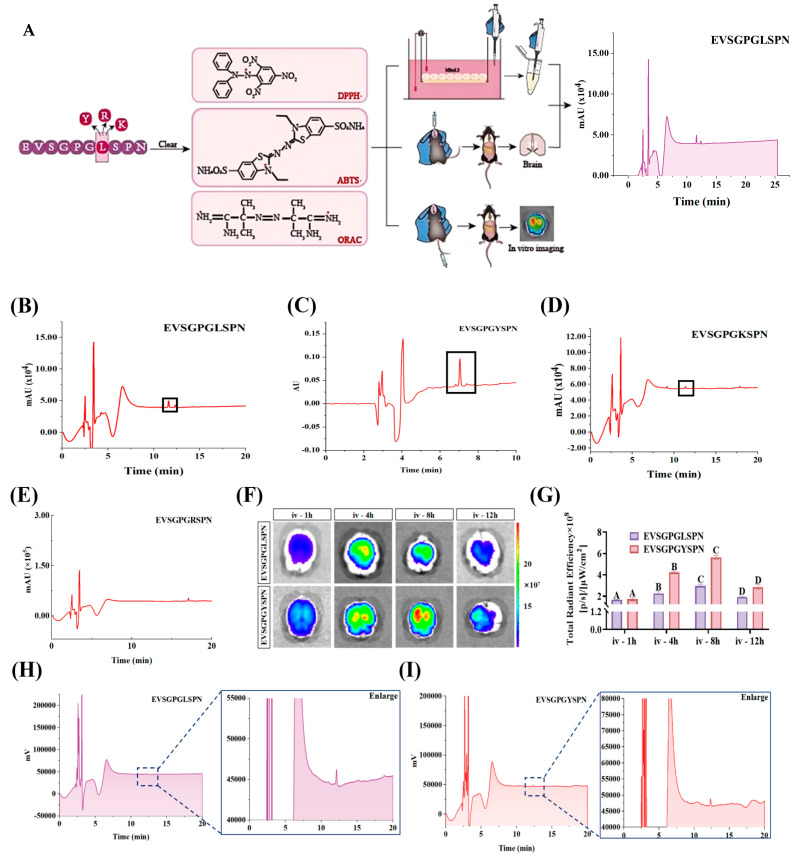
Determination of apparent permeability of peptides. (**A**) Flowchart, (**B**–**E**) determination of apparent permeability of EV peptides in Transwell, (**F**,**G**) Ex vivo imaging of tail vein injection of EV peptides, (**H**,**I**) detection of accumulated concentration of EV peptides in mouse brain after gavage injection. All experiments were conducted in triplicate (*n *= 3). Statistical significance was considered at *p* < 0.05. Dissimilar letters represent significant differences between groups, whereas identical letters indicate no significant difference.

To investigate the BBB penetration ability of EV peptides, an in vitro BBB model was established (Figure 4A). A dense monolayer of bEnd.3 cells were seeded in a Transwell upper chamber with an average pore size of 0.4 µm (Figure S1A). BBB integrity was assessed by measuring TEER (Figure S1B), and the concentration of EV peptides in the Transwell lower chamber was detected utilizing RP-HPLC. After 3 days of culture, the TEER value of the bEnd.3 cells in the upper chamber were 66.63 ± 3. 67 Ω·cm^2^. After 11 days, the TEER value increased to 229.52 ± 4.18 Ω·cm^2^. At 14 days, the TEER values plateaued, indicating the successful construction of the BBB in vitro model. These results align with Han’s report [33], which also used TEER values above 200 Ω·cm^2^ for their BBB model. The apparent Papp values of EVSGPGLSPN and EVSGPGKSPN at 4 h were 3.78 ± 0. 40 × 10^−6^ cm/s and 1.36 ± 0. 15 × 10^−6^ cm/s, respectively (Figure 4B,C). This is significantly lower than the Papp value of EVSGPGYSPN of 8.10 ± 0.34 × 10^−6^ cm/s (*p* < 0.05) (Figure 4D). However, the presence of EVSGPGRSPN in the Transwell lower compartment was nearly undetectable, indicating that the BBB may restrict its transport to the brain. (Figure 4E) These findings suggest that EVSGPGYSPN penetrates tight bEnd.3 cell monolayers more effectively than EVSGPGLSPN and EVSGPGKSPN. This may be attributed to variations in amino acid sequences among P, which consequently alter changes in the secondary structure of the peptides. The R is likely constrained by the P in the peptide, which in turn alters its charge distribution and reduces its lipophilicity. This, combined with its potentially suboptimal interaction with transporters on brain endothelial cells, ultimately leads to the low BBB penetration rate observed for EVSGPGRSPN.

Efficient antioxidant-based delivery across the BBB is effective in alleviating brain diseases, such as AD [5]. For example, both the 8-hydroxyquinoline derivative 5b and acetylcholinase inhibitor 4u penetrate the in vitro BBB model, with ORAC values of 2.63 Trolox Eq and 3.28 Trolox Eq, respectively [49,50]. These compounds have been reported as the most promising therapeutic agents for AD owing to their neuroprotective properties. However, food-derived bioactive peptides, including soybean dipeptides Gly-Sar, GP, and YP, penetrated the in vitro BBB model with apparent permeability of 7.60 ± 1.29 µL/g·min, 3.49 ± 0.66 µL/g·min, and 3.53 ± 0.74 µL/g·min, respectively, though their antioxidant activity remains unknown. However, our study showed that the strong BBB-penetrating ability of walnut-derived peptides EVSGPGYSPN and TWLPYPR was accompanied by their high antioxidant activity. This is likely because peptides containing aromatic or hydrophobic amino acids can enhance free radical scavenging activity by promoting interactions with lipids or acting as proton or hydrogen donors. However, the correlation between the brain-targeting ability and antioxidant activity of bioactive peptides still requires further exploration, particularly the interaction between aromatic amino acids and phospholipid bilayers.

We then evaluate the ability of EVSGPGLSPN and EVSGPGYSPN to cross the BBB in vivo via a single tail vein injection. The ex vivo imaging results, presented in Figure 4F,G, show that both peptides demonstrated strong Rhodamine B fluorescence in the brain within 4 h of tail vein injection, which steadily increased to the peak values of 5.63 ± 0.20 × 10^8^ [p/s]/[µW/cm^2^] for EVSGPGYSPN and 2.95 ± 0.18 × 10^8^ [p/s]/[µW/cm^2^] for EVSGPGLSPN at 8 h. The fluorescence was sustained at 12 h, indicating that the peptides efficiently crossed the BBB, accumulated, and were retained in the brain. Subsequently, we evaluated the effective concentrations of EVSGPGLSPN and EVSGPGYSPN in mouse brains. The transport of these two peptides across the mouse BBB was analyzed in brain homogenates utilizing the RP-HPLC technique previously described. Figure 4H,I show the concentrations of EVSGPGLSPN and EVSGPGYSPN accumulated in the mouse brain reached 0.25 ± 0.11 µg/g and 1.25 ± 0.91 µg/g, respectively, following oral administration of the EV peptides for 10 h. In contrast, the maximum concentration of eveltivir, a drug used to treat brain disease, in the brain is only 46.5 ± 3.5 ng/g [51]. These findings further demonstrate the brain-targeting capability of EVSGPGYSPN. This observation strongly suggests that the decapeptide compounds EVSGPGLSPN and EVSGPGYSPN may be transported across the BBB in the blood–brain direction.

BBB-penetrating peptides are crucial in preventing and treating brain diseases [52]. Previous research has demonstrated that these peptides can reverse the pathological features of brain disease, including neuroinflammation [53], oxidative stress [54], memory impairment [55], abnormal microglia activation [56], Aβ accumulation, and abnormal Tau protein phosphorylation [57]. However, their limited ability to penetrate the BBB and potential adverse effects hinder their clinical translation in AD [58]. Therefore, bioactive peptides derived from food show great potential because of their safety and reliability. To date, Scientists have proposed various strategies to enhance the delivery efficiency of bioactive peptides to the brain. Among these, structural modifications to peptides, including cyclization, lipidation, and methylation, have been widely explored. Because the amino acid residues in the side chain of linear peptides are easily isomerized and have low receptor selectivity, they are easily degraded by lysosomes in the BBB. In contrast, polycyclic peptides can form stable complexes with BBB transporters or receptors by restricting their conformation, thereby facilitating BBB penetration. However, the role of structure–activity relationships in overcoming BBB complexity to achieve targeted brain delivery remains inadequately understood.

Peptides with high benzene cyclic hydrogen signals usually have high hydrophobicity, which facilitates their interaction with the phospholipid bilayer on the BBB. Therefore, many peptides containing benzene rings have been shown to fuse with the cell membrane to penetrate the BBB, such as Met-enkephalin and Leu-enkephalin. Nonetheless, they are readily degraded by proteases in the gastrointestinal tract and plasma, resulting in poor BBB permeability and often requiring modifications such as glycosylation to promote their penetration into the BBB [59]. Strong BBB penetration of peptides EVSGPGLSPN and EVSGPGYSPN may be related to their stable gastrointestinal digestive capacity and good plasma half-life. A notable example includes polyarginine peptides such as R5, R7, and R9, whose transmembrane ability is positively correlated with arginine content [60]. This is attributed to the hydrogen bond formed between the guanidino group, with a pKa of approximately 12 on its side chain, and the phosphate group [61]. However, in this study, we found that the peptides based on L and Y structure also had BBB penetration ability, while the structure of peptides with tyrosine sandwiched between proline conferred enhanced targeted delivery ability. Conversely, the R-based peptides failed to penetrate the in vitro BBB model. These findings hold significant pharmaceutical and chemical implications, as approximately 78% of peptides known to alleviate cognitive deficits or exhibit neuroprotective properties contain aromatic amino acid residues, with 44.4% of them containing Y [62]. Compared to other naturally occurring amino acids, P has a unique pyrrolidine ring structure and a fixed ψ angle. When present in large quantities within the amino acid sequence, it can form a left-handed, extended, specific secondary structure known as polyproline II (PII). The incorporation of PII directly affects the internalization ability of peptides [62]. Such as peptide GLRILLLKV-NH_2_ [63]. These structural features enable penetration of the BBB and neuroprotective bifunctionalization. To sum up, secondary structure analysis shows that amino acid composition affects the conformation of the peptide chain, which may be the key factor affecting antioxidant function activity.

### 3.5. Evaluation of the Biostability and Neuroprotective Function of EV Peptides

#### 3.5.1. In Vitro Simulation of Gastrointestinal Digestion of EV Peptides

**Figure 5:**
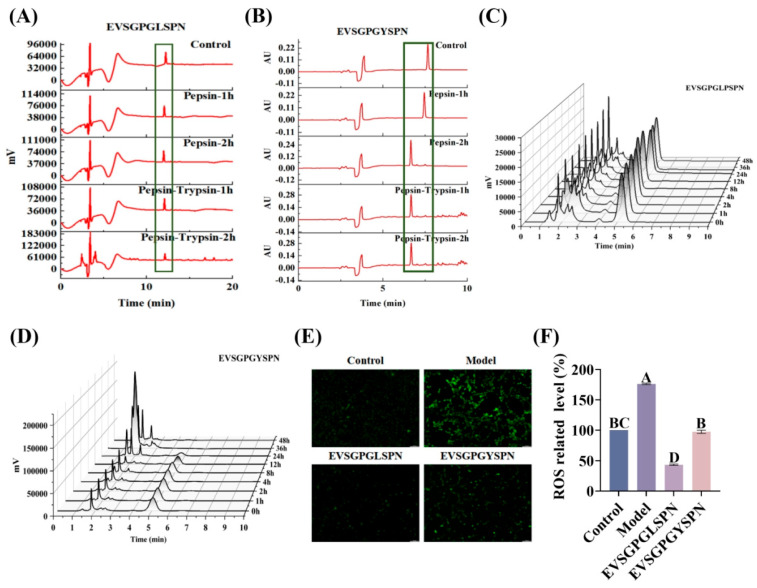
In vivo digestion and neuroprotection of EV peptides. (**A**,**B**) Gastrointestinal digestion simulation, (**C**,**D**) half-life detection of EVSGPGLSPN and EVSGPGYSPN in plasma, (**E**,**F**) changes in ROS levels in HT22 cells. All experiments were conducted in triplicate (*n* = 3). Different letters indicate statistically significant differences (*p* < 0.05).

While these BBB-penetrating peptides demonstrate good penetration ability and antioxidant activity in vitro, they must withstand proteolytic enzyme degradation in the gastrointestinal tract and pass through blood circulation before crossing the highly selective BBB for targeted brain delivery. To evaluate if the peptides are resistant to gastrointestinal digestion, EVSGPGLSPN and EVSGPGYSPN were incubated with pepsin and trypsin, and the resulting peptides were separated using RP-HPLC. Figure 5A,B shows that both peptides exhibited peptide ions of corresponding mass at each time point during pepsin and trypsin digestion. The gastrointestinal digestive system recovery rate was 19.52 ± 3.33% and 13.99 ± 0.02%, respectively, indicating their ability to resist gastrointestinal digestion.

The gastrointestinal tract serves as the first interface between the human body and the external environment. Peptides are prone to hydrolysis in the digestive tract, consequently reducing their bioavailability and potential biological activity. For example, the anticancer peptides LYSPH and PSYLNTPLL, although capable of penetrating both cells and the BBB, accumulate in the brain to a limited extent owing to their resistance to gastrointestinal digestion [64]. Therefore, stable gastrointestinal digestion is the first step in achieving brain-targeted delivery of functional factors. The walnut-derived peptide EVSGPGYSPN identified in this study exhibits superior BBB penetration and resists gastrointestinal digestion, enabling stable entry into the bloodstream.

#### 3.5.2. Half-Life of Plasma

The short plasma half-life of BBB-penetrating peptides limits their in vivo efficacy [59]. Consequently, we predicted the percentage and oral bioavailability of the peptides EVSGPGLSPN and EVSGPGYSPN over time using in vitro plasma models. The degradation curves are presented in Figure 5C,D. After 2 h, the peptides EVSGPGLSPN and EVSGPGYSPN exhibited good stability in plasma, with retention rates of 99.00 ± 0.75% and 92.42 ± 0.74%, respectively. However, after 4 h, EVSGPGYSPN began to degrade gradually in plasma. At 24 h, only 24.29 ± 1.94% remained, while EVSGPGLSPN remained at 95.62 ± 0.64%. By 36 h, EVSGPGYSPN was completely undetectable in plasma, indicating complete degradation. In contrast, EVSGPGLSPN still exhibited a retention rate of 94.77 ± 0.64% at 48 h. These findings indicate that EVSGPGLSPN has superior stability in plasma compared to that of EVSGPGYSPN, as the in vitro plasma half-life of EVSGPGLSPN exceeds 48 h.

Bioactive peptides must remain stable in the blood circulation to be able to participate in physiological activities in the brain. However, during circulation, they can be degraded by various plasma enzymes, such as trypsin and plasmin [65]. The plasma stability of various BBB-penetrating peptides has been reported; however, most of them need to be administered orally or injected multiple times. Moreover, some even require carriers for embedded delivery or modification to achieve sufficient accumulation in the brain [66]. For instance, the neuropeptide TLQP-21, which contains 4 Rs, rapidly degrades after 60 min in vitro plasma. Consequently, this neuropeptide fails to reach the brain to exert neuroprotective effects when administered via intravenous injection [67]. The BBB-penetrating peptide H-Tyr-D-Ala-Gly-MePhe-Gly-ol exhibits a plasma half-life of only 9.2 ± 2.1 min, but this can be extended to 6.9 ± 2.3 h by embedding and delivering it with GSH-PEG liposomes [68]. Shorter half-lives are insufficient for delivering adequate concentrations of functional factors to target tissues [69]. In contrast, the retention rate of EVSGPGYSPN reaches 50.96 ± 0.64% after 12 h (Table 3). This indicates a half-life of up to 12 h, which is higher than several current BBB-penetrating peptides. However, some bioactive peptides are stable in plasma, although their BBB penetration ability has not been verified. For instance, EI Mubarak et al. found that the peptide PCK3145 derived from prostate secreted protein 94 exhibited a peptide retention rate exceeding 98% after incubation with human plasma for 24 h [70]. This is comparable to the peptide EVSGPGLSPN. However, there are still variations in the in vitro determination of plasma half-life across different species. Therefore, future studies are warranted to verify the plasma stability of peptides EVSGPGLSPN and EVSGPGYSPN across various species as well as in vivo.

The brain selectively absorbs nutrients from the blood while maintaining homeostasis by excluding various compounds and metabolites through the BBB system, which separates the circulating blood from the brain. This limits the accumulation of neuroprotective factors, including peptides, in the brain. To effectively target the central nervous system deep within the brain, these functional factors must remain stable in both the gastrointestinal tract and bloodstream long enough to penetrate the BBB and accumulate in the brain. Several studies suggest foodborne peptides as potential treatments for brain diseases, but they lack practical parameters for clinical application, with poor in vivo stability and ineffective brain accumulation limiting their therapeutic effectiveness. Our identified BBB-penetrating peptide EVSGPGLSPN demonstrated high stability in blood circulation, with a decrease of only 2.23% at 48 h. While EVSGPGYSPN is less stable than EVSGPGLSPN during gastrointestinal digestion and in blood circulation, its effective concentration in the brain is significantly higher (*p* < 0.05). This may be attributed to the higher affinity of EVSGPGYSPN for transporters on the BBB, which facilitates its efficient crossing before degradation, thereby compensating for its lower plasma stability compared to EVSGPGLSPN. Furthermore, it is highly plausible that certain degradation products of EVSGPGYSPN in plasma may also possess the ability to cross the BBB, working synergistically with the intact peptide to support brain health—a hypothesis worthy of more in-depth investigation in the future. Greater emphasis should also be placed on developing advanced delivery strategies to enhance the stability of EVSGPGYSPN and explore its potential for clinical applications.

#### 3.5.3. Neuroprotective Properties

To investigate the neuroprotective effects of these BBB-penetrating peptides, HT22 cells were exposed to 200–1600 µM H_2_O_2_ for 2 h (Figure S2), and cell viability was determined utilizing an MTT assay. As the H_2_O_2_ concentration increased, cell viability gradually declined. At 800 µM H_2_O_2_, the cell survival rate dropped to 50.05 ± 1.50%, indicating successful modeling. Consequently, a conditionally lesioned HT22 cell model using 800 µM for 2 h was selected. HT22 cells were then treated using the modeling conditions established in the previous experiments. HT22 cells were treated with 100 µM of EVpeptides for 24 h, followed by exposure to H_2_O_2_ for 2 h. Cell viability was then measured employing the MTT assay. Compared with the model group (49.23 ± 1.00%), the cell viability of the peptides EVSGPGYSPN and EVSGPGLSPN significantly improved, with values of 86.88 ± 3.18% and 78.63 ± 0.86%, respectively (*p* < 0.05). This indicates that peptides with high antioxidant capacity exhibit stronger neuroprotective effects and confirms that the BBB-penetrating peptides identified can mitigate H_2_O_2_-induced injury in HT22 cells.

Next, we used DCFH-DA fluorescent probes to detect the effects of the peptides EVSGPGLSPN and EVSGPGYSPN on ROS levels in HT22 cells. Subsequently, we explored their protective ability against apoptosis (Figure 5E). The relative content of ROS in HT22 cells in the H_2_O_2_ group increased to 176.42 ± 1.19%. After treatment with 100 um EVSGPGLSPN and EVSGPGYSPN, ROS significantly decreased to 43.49 ± 1.08% (*p* < 0.01) and 110.46 ± 15.16% (*p* < 0.05), respectively. This result indicated that EVSGPGLSPN and EVSGPGYSPN reduced H_2_O_2_-induced ROS, thereby protecting HT22 cells. Furthermore, EVSGPGYSPN significantly reduced early cell apoptosis and late cell apoptosis caused by H_2_O_2_ (Figure 5F).

### 3.6. In Vitro BBB Penetration Ability and Secondary Structure Validation of TWLPYPR and YVPFPYP

**Figure 6:**
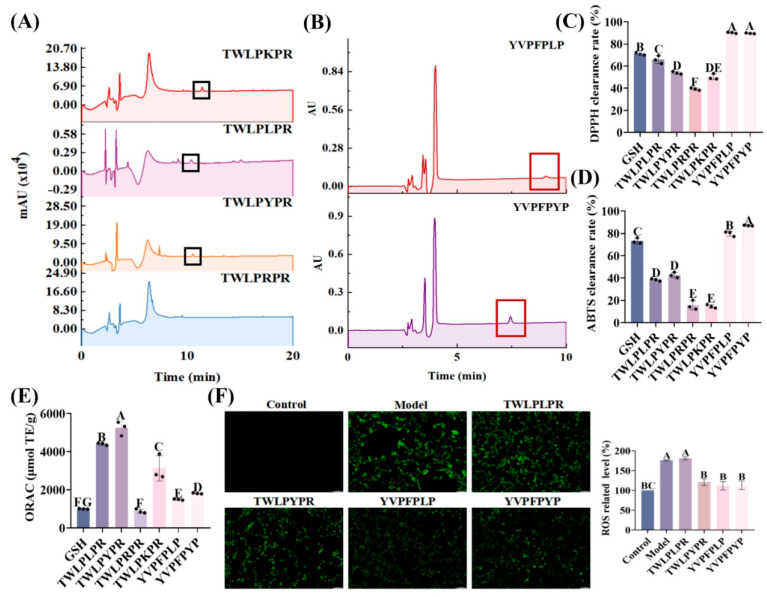
Validation was performed with walnut peptides TWLPLPR and YVPFPLP. The ability of the TW peptide and YV peptide (**A**,**B**) to penetrate the BBB model in vitro, (**C**–**E**) ability to scavenge ABTS, DPPH and oxygen free radicals test, (**F**) inhibitory effect on ROS levels in H_2_O_2_-induced HT-22 cells. Statistical significance was considered at *p* < 0.05. Dissimilar letters represent significant differences between groups, whereas identical letters indicate no significant difference.

To show that Y is the main reason for the enhanced peptide penetration rate, we substituted the fifth amino acid site of walnut peptide TWLPLPR with Y, K and R, and replaced the sixth amino acid site of walnut peptide YVPFPLP with Y to explore the trans-BBB transport ability of these peptides. As shown in Figure 6A, after 4 h, TWLPKPR and TWLPLPR penetrated the in vitro BBB model with Papp values of 3.45 ± 0.40 × 10^−6^ cm/s and 6.85 ± 0.51 × 10^−6^ cm/s, respectively, which were significantly lower than that of TWLPYPR of 7.06 ± 0.51 × 10^−6^ cm/s (*p* < 0.05). In contrast, the presence of TWLPRPR was also completely undetectable in the lower compartment (we summarized the apparent permeability of these BBB-penetrating peptides in Table 2). Similarly, walnut-derived peptide YVPFPYP penetrated the in vitro BBB model with a Papp value of 3.62 ± 0.47 × 10^−6^ cm/s, which was significantly higher than the Papp value of walnut peptide YVPFPLP of 9.99 ± 0.05 × 10^−7^ cm/s (Figure 6B). This validates our previous conclusion that walnut-derived peptides based on the Y structure have the strongest BBB penetration ability. And these peptides have the ability to inhibit the increase in ROS level in HT22 cells induced by H_2_O_2_ (Figure 6F).

The antioxidant properties and neuroprotective functions of these peptides were subsequently analyzed. As shown in Figure 6C, among the TW peptides, TWLPLPR showed the highest DPPH free radical scavenging rate (62.63 ± 3.55%), which was not significantly different from that of GSH (70.98 ± 1.09%, *p* > 0.05). This may be attributed to the inherently low molecular weight of TWLPLPR, as it is the smallest among the TW peptides. Similarly, the DPPH clearance of YVPFPLP (90.22 ± 0. 55%) was slightly higher than that of YVPGPYP (89.68 ± 0.37%), although the difference was not statistically significant (*p* > 0.05). However, both peptides exhibited significantly higher DPPH clearance rates than did GSH (70 ± 1.09%, *p* < 0.05). In contrast to the DPPH results, the ABTS free radical scavenging ability of TWLPYPR (42.64 ± 2.46%) was significantly higher than that of TWLPLPR (38.41 ± 1. 01%, *p* < 0.05). Additionally, the ABTS free radical scavenging ability of YVPFPLP and YVPFPYP was 79.76 ± 2.20% and 86.99 ± 0.34%, respectively (Figure 6D), both significantly higher than that of GSH (73.20 ± 2.54%, *p* < 0.05). The ORAC assay further revealed that TWLPYPR and YVPFPYP exhibited clearance capacities of 5237.92 ± 359.72 µmol TE/g and 1813.30 ± 28.74 µmol TE/g, respectively, significantly higher than that of TWLPLPR and YVPFPLP (*p* < 0.05) (Figure 6E). Overall, these results indicate that tyrosine-based peptides had stronger ABTS free radical scavenging ability and oxygen free radical scavenging ability, whereas leucine-based peptides had stronger DPPH free radical scavenging ability.

**Figure 7:**
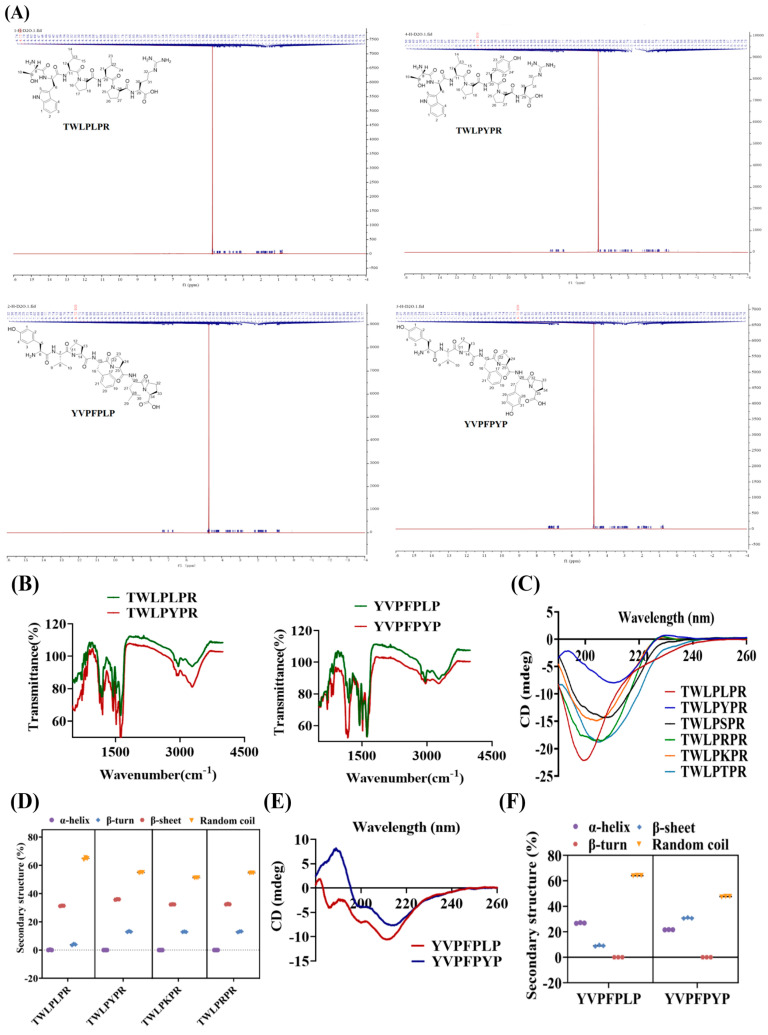
Comparison of the Secondary Structures of the TW and YV Peptides. (**A**) NMR test, (**B**) FTIR, and (**C**–**F**) CD. All experiments were conducted in triplicate (*n *= 3).

As previously mentioned, we hypothesize that the benzene ring hydrogen signal plays a key role in facilitating the transport of these walnut-derived peptides across the BBB. To test this hypothesis, we conducted H-NMR and Fourier infrared spectroscopy to verify the secondary structures of the peptides TWLPLPR, TWLPYPR, YVPFPLP and YVPFPYP. As shown in Figure 7A, there are four aromatic hydrogen signals and five methyl signals in the hydrogen spectrum of TWLPLPR, while there are eight aromatic hydrogen signals and three methyl signals in TWLPYPR. This difference in characteristic hydrogen signal corresponds exactly to the isopropyl substitution of TWLPLPR at the C21 position versus the p-hydroxyphenyl substitution of TWLPYPR at the C21 position. FTIR analysis further confirmed that TWLPYPR exhibited a stronger benzene ring skeleton vibration peak. Similarly, the primary difference between the hydrogen spectra of YVPFPLP and YVPFPYP was the nature of the substitution at C-27—either an isopropyl or p-hydroxyphenyl group. The hydrogen spectrum of YVPFPLP revealed nine aromatic hydrogen signals and four methyl signals, whereas YVPFPYP exhibited thirteen aromatic hydrogen signals and two methyl signals (Figure 7B). FTIR analysis further showed that the vibration peak of the benzene ring skeleton of YVPFPYP is stronger, with a distinct absorption peak at 815 cm^−1^, indicating that YVPFPYP has more para-substituted benzene ring structures than YVPFPLP. These findings further confirm that walnut-derived peptides with high benzene ring hydrogen signals have stronger BBB penetration ability.

The circular dichroism spectrum (Figure 7C,D) revealed that TWLPLPR and TWLPYPR contained 64.73 ± 0.80% and 51.13 ± 0.12% irregular coil structures, respectively, which were significantly higher than the β-sheet content (31.27 ± 0.15% and 35.83 ± 0.21%, *p* < 0.05). Similarly, YVPFPLP and YVPFPYP contained 64.07 ± 0.15% and 47.63 ± 0.29% irregular coil structures, respectively, which were significantly higher than their α-helix, β-turn, and β-sheet contents (*p* < 0.05; Figure 7E,F). Notably, BBB-penetrating peptides showed the highest content of irregular coil. These results suggest that peptide folding and conformational changes enhance their compatibility with the transport channels or binding sites of the BBB, likely due to increased benzene ring hydrogen signaling. This adaptation facilitates interactions with BBB transporters or receptors, thereby promoting BBB penetration efficiency.

## 4. Conclusions

In summary, we successfully obtained a walnut-derived peptide, EVSGPGYSPN, which exhibits high antioxidant activity (ABTS radical scavenging activity and ORAC activity), strong neuroprotective capacity, and efficient transport across the BBB. This enhanced performance stems from the substitution of L with Y in the parent peptide EVSGPGLSPN, likely due to the introduction of Y leading to reduced methyl signals, enhanced aromatic ring hydrogen signals, and increased β-sheet content. Similarly, two other walnut-derived peptides, TWLPYPR and YVPFPYP, which underwent analogous substitutions, also demonstrated the same superior properties. Interestingly, in all cases, the substitution occurred between two proline residues—a specificity that warrants further investigation. These findings provide valuable insights and potential strategies for screening and designing high-performance peptides. In particular, they introduce novel peptide structures for the development of functional compounds aimed at preventing and alleviating brain disorders. Furthermore, we systematically validated the stability of both EVSGPGLSPN and EVSGPGYSPN under gastrointestinal digestion and in the circulatory system.

## Figures and Tables

**Table 1:** EV peptide molecule docking.

Sequence	Binding Energy (kcal/mol)	Ki (Kd) (298 K, nM)	PI	GRAVY	Solubility	Number of Net Charges at pH = 7.0
EVSGPGYSPN	−18.3	2,380,115.19	9.36	−0.93	Hydrophobic	0.98
EVSGPGKSPN	−17.7	153,583.29	9.71	−1.23	Hydrophilic	0.98
EVSGPGRSPN	−17.4	223,109.42	10.56	−1.29	Hydrophilic	0.98
EVSGPGFSPN	−17.3	18,455.9	6.36	−0.56	Hydrophobic	−0.02
EVSGPGSSPN	−17.3	17,249.74	6.36	−0.92	Hydrophilic	−0.02
EVSGPGTSPN	−17.3	220,858.95	6.36	−0.91	Hydrophilic	−0.02
EVSGPGASPN	−17.2	56,866.77	6.36	−0.66	Hydrophilic	−0.02
EVSGPGWSPN	−17.1	62,194.35	6.36	−0.93	Hydrophobic	−0.02
EVSGPGDSPN	−16.9	25,356.733	4	−1.19	Hydrophilic	−1.02
EVSGPGVSPN	−16.9	21,450.88	6.36	−0.42	Hydrophilic	−0.02
EVSGPGMSPN	−16.7	11,211.37	6.36	−0.65	Hydrophilic	−0.02
EVSGPGNSPN	−16.4	92,825.22	6.36	−1.19	Hydrophilic	−0.02
EVSGPGGSPN	−16.4	10,857.16	6.36	−0.88	Hydrophilic	−0.02
EVSGPGHSPN	−16.4	98,813.73	7.56	−1.16	Hydrophilic	0.21
EVSGPGISPN	−16.3	66,543.2	6.36	−0.39	Hydrophilic	−0.02
EVSGPGCSPN	−16.2	10,147.6	8.56	−0.59	Hydrophilic	0.94
EVSGPGQSPN	−16.2	55,725.34	6.36	−1.19	Hydrophilic	−0.02
EVSGPGLSPN	−16.2	5058.6	6.36	−0.46	Hydrophilic	−0.02
EVSGPGESPN	−15.9	25,571.86	4.1	−1.19	Hydrophilic	−1.02
EVSGPGPSPN	−15.4	25,701.81	6.36	−1	Hydrophilic	−0.02

**Table 2:** BBB penetration ability of EV, TW and YV peptides.

Sequence	Papp (cm/s)	Brain Accumulation Concentration (cm/s)
EVSGPGLSPN	3.78 ± 0.40 × 10^−6^	0.25 ± 0.11 µg/g
EVSGPGYSPN	8.10 ± 0.34 × 10^−6^	1.25 ± 0.91 µg/g
EVSGPGKSPN	1.36 ± 0.15 × 10^−6^	
EVSGPGRSPN	-	
TWLPLPR	6.85 ± 0.51 × 10^−6^	
TWLPYPR	7.06 ± 0.51 × 10^−6^	
TWLPKPR	3.45 ± 0.40 × 10^−6^	
TWLPRPR	-	
YVPFPLP	9.99 ± 0.05 × 10^−7^	
YVPFPYP	3.62 ± 0.47 × 10^−6^	

**Table 3:** Retention rate of EVSGPGLSPN and EVSGPGYSPN in plasma at different times.

Time	EVSGPGLSPN	EVSGPGYSPN
0 h	100%	100%
1 h	99.75 ± 0.35%	96.64 ± 0.53%
2 h	99.00 ± 0.75%	92.42 ± 0.74%
4 h	98.13 ± 0.06%	80.50 ± 0.65%
8 h	97.86 ± 0.49%	58.64 ± 0.52%
12 h	96.86 ± 0.50%	50.96 ± 0.64%
24 h	95.62 ± 0.64%	24.29 ± 1.94%
36 h	94.96 ± 0.36%	0%
48 h	94.77 ± 0.64%	0%

## Data Availability

The original contributions presented in this study are included in the article/Supplementary Material. Further inquiries can be directed at the corresponding author.

## Supplementary Materials

The following supporting information can be downloaded at: https://www.mdpi.com/article/10.3390/foods14213744/s1, Table S1: Molecular weights of EV peptides and TW peptides. Figure S1: Variation of resistance value. Figure S2: Neuroprotective properties of BBB-penetrating peptides in vitro. Figure S3: (A) Blank plasma RP-HPLC detection, (B) EVSGPGLSPN sequence control detection, (C) EVSGPGYSPN sequence control. All experiments were conducted in triplicate (n = 3).
