# Supplementary material for: Structural Engineering of Tyrosine-Based Neuroprotective Peptides: A New Strategy for Efficient Blood–Brain Barrier Penetration
Source: Foods. 2025 Oct 31;14(21):3744. doi: 10.3390/foods14213744 (PMC12610161; doi:10.3390/foods14213744)
Supplement: Supplementary file 1 [file foods-14-03744-s001.zip › foods-3922617-supplementary.pdf]

Table S1 Molecular weights of EV peptides and TW peptides

Sequence	Molecular weight
EVSGPG**L**SPN	956.01
EVSGPG**Y**SPN	1006.02
EVSGPG**K**SPN	971.02
EVSGPG**R**SPN	998.48
TWLP**L**PR	882.06
TWLP**Y**PR	932.07
TWLP**K**PR	897.07
TWLP**R**PR	925.08
YVPFP**L**P	831.99
YVPFP**Y**P	882.01

Figure S1

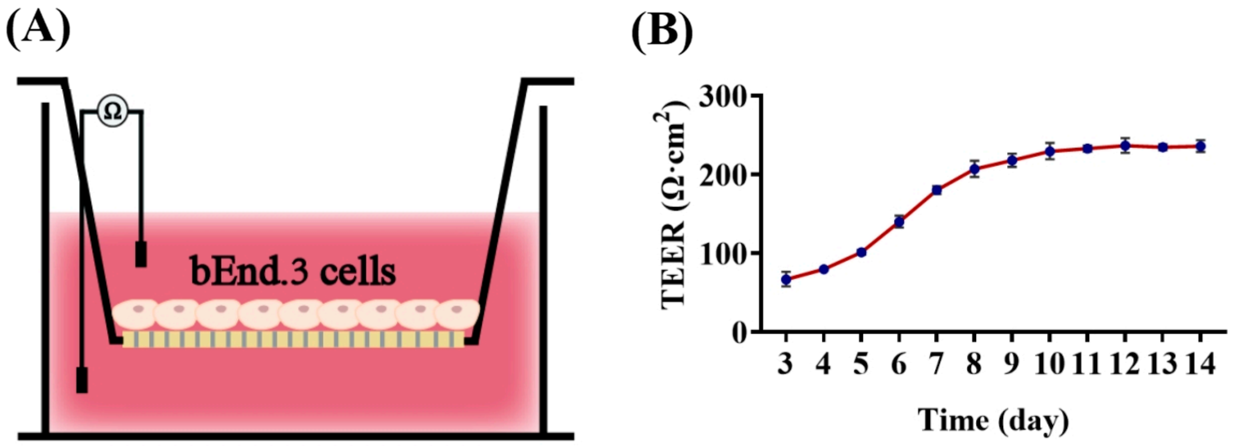

Fig. S1 Variation of resistance value.

Figure S2

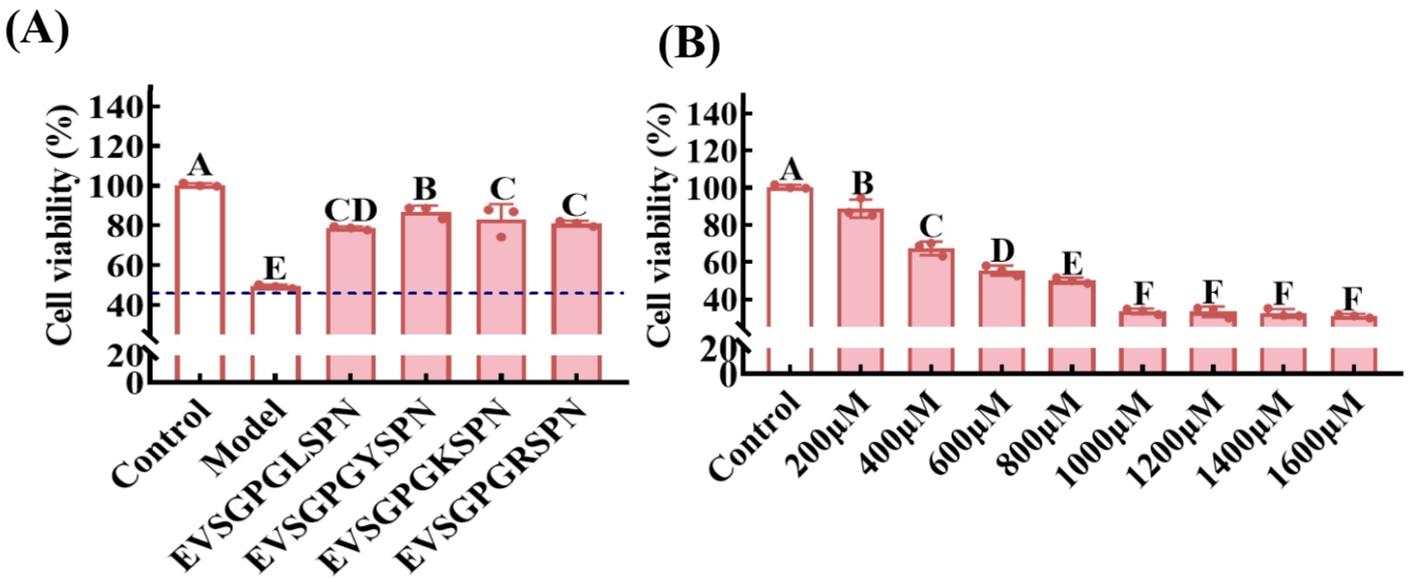

Fig.S2 Neuroprotective properties of BBB-penetrating peptides in vitro. (A) MTT screening for optimal concentration of H_2O_2, (B) determination of viability of H_2O_2-injured HT22 cells by BBB penetrating peptide. All experiments were conducted in triplicate ($n = 3$). Different letters indicate statistically significant differences ($p < 0.05$).

Figure S3

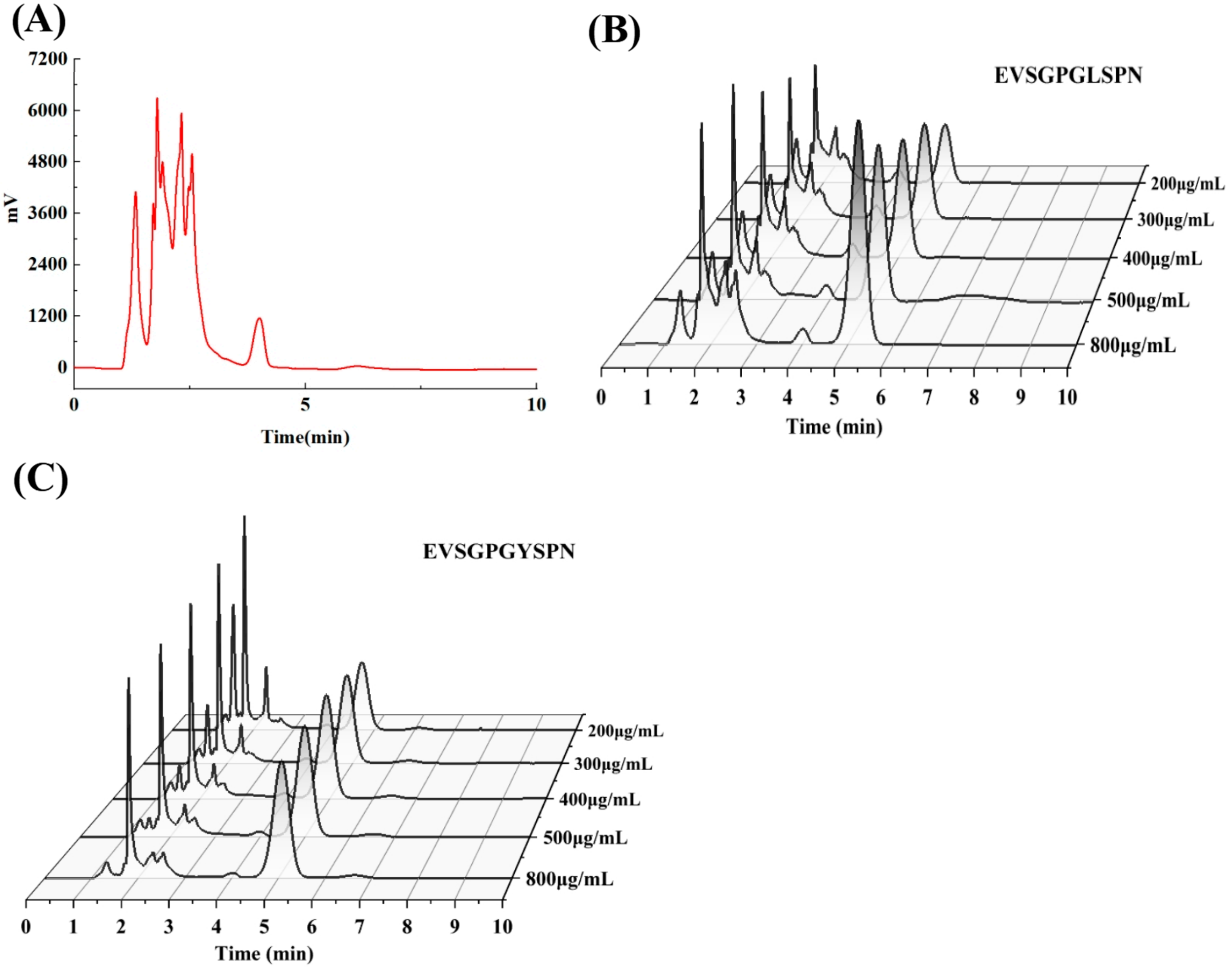

Fig.S3 (A) Blank plasma RP-HPLC detection, (B) EVSGPGLSPN sequence control detection, (C) EVSGPGYSPN sequence control. All experiments were conducted in triplicate (*n* = *3*). Different letters indicate statistically significant differences ($p < 0.05$).
